# Recent advances in hematopoietic stem cell transplantation

**DOI:** 10.12688/f1000research.11233.1

**Published:** 2017-06-12

**Authors:** Maxim Norkin, John R Wingard

**Affiliations:** 1 Department of Medicine, University of Florida, Gainesville, Florida, 32610-0277, USA

**Keywords:** Hematopoietic stem cell, hematopoietic stem cell transplant, HCT, graft versus host, GVHD, transplant toxicity

## Abstract

Hematopoietic cell transplantation (HCT), once used as a last-resort therapy, is now considered a lifesaving procedure for thousands of patients with life-threatening diseases worldwide and is frequently used early in the course of treatment for diseases destined to be uncontrollable by non-HCT therapies. Incremental advances leading to reduction of post-transplant morbidity and mortality by better control of graft versus host disease (GVHD), infections, and regimen-related toxicities, coupled with greater donor options, not only significantly increased the utilization and success of this procedure but also allowed many of these patients to enjoy healthy and productive lives after HCT. Emerging concepts in the field are now focused on the expansion of available donor options, further reduction of transplant-related toxicity, and decrease in post-transplant relapse.

## Introduction

**Figure 1.**
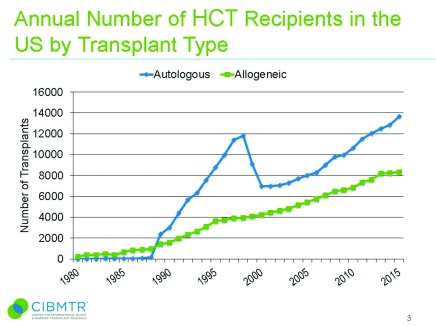
The estimated annual numbers of hematopoietic stem cell transplant (HSCT) recipients in the United States of America according to the Center for International Blood and Marrow Transplant Research data. The figure has been reproduced with permission from CIBMTR. D’Souza A, Zhu X (2016) ^8^. https://www.cibmtr.org/ReferenceCenter/SlidesReports/SummarySlides/pages/index.aspx

When the first hematopoietic cell transplant (HCT) was performed six decades ago, it was used as a last-resort therapy in an attempt to deliver high doses of radiation and chemotherapy to patients with incurable malignancies ^1^. Since then, HCT has become a lifesaving procedure for millions of patients. In the US alone, there were almost 14,000 autologous (auto)-HCTs and more than 8,000 allogeneic (allo)-HCTs performed in 2015, and the number of HCTs is steadily increasing ( Figure 1).

The success of HCT has resulted from continuous advances in the field. The recent advances of greatest impact can be grouped into three major categories: 1) reduction of transplant-related morbidity and mortality, 2) expansion of donor options, and 3) reduction of post-transplant relapse.

## Reduction of transplant-related morbidity and mortality

There have been substantial reductions in mortality after allo-HCT owing to a decrease in organ damage, better prophylaxis and treatment strategies for infectious complications, and improved techniques for the prevention and management of graft versus host disease (GVHD) in the 2003–2007 period, as noted in one study, when compared with the 1993–1997 period ^2^. Importantly, this decline in mortality occurred despite the fact that older patients and patients with more comorbidities were undergoing allo-HCT in increasing numbers in recent decades. This was an important change in practice because most hematologic malignancies occur in the elderly and formerly most were excluded from consideration for allo-HCT. Better prevention and control of infections, particularly cytomegalovirus (CMV) and invasive fungal infections, is one of the most important contributions leading to improved HCT outcomes in recent years ^2^. Antimicrobial prophylaxis or pre-emptive therapies using broad-spectrum antibiotics and potent oral antiviral and antifungal agents are now considered as a standard of care worldwide. Widely adopted fluconazole prophylaxis led to marked reduction of invasive candidiasis after HCT and improved survival ^3^. The development of sensitive radiologic studies, microbiologic and histopathologic techniques, and fungal biomarker assays now allows early diagnosis of invasive mold fungal infections and facilitates prompt institution of therapy that has resulted in improved treatment outcomes ^4^. Most recently, the introduction of novel agents with reduced toxicity and broad-spectrum antifungal activity such as posaconazole ^5^ and isavuconazole ^6^ both for prophylaxis and therapy for mold infections has led to decreased use of older, more toxic agents such as amphotericin B-based antifungal drugs. Advances in molecular diagnostic testing for CMV viremia and acceptance of pre-emptive antiviral therapy as a mainstay strategy for the prevention of symptomatic CMV disease after HCT has been shown to be highly effective in minimizing the risk of CMV end-organ disease ^2, 7^.

GVHD remains one of the main complications after allo-HCT. Despite very intensive research, no major advances in the medical management of GVHD were made during the last two decades. Significant heterogeneity and a very complex pathogenesis of the disease as well as lack of well-defined specific therapeutic targets may explain this scarcity of proven success of novel drug therapies in patients with GVHD. However, over the last two decades, the decrease in frequency and severity of GVHD has led to a reduction in transplant-related mortality ^2^. This can be explained by changes in clinical practice associated with more effective prevention of severe acute and chronic GVHD. Selection of better-matched unrelated donors due to the use of high-resolution human leukocyte antigen (HLA) typing, increased use of non-myeloablative conditioning, and decreased use of total body irradiation in recent years can be associated with a reduction in the severity of acute GVHD ^9^. Increased frequency of post-transplant cyclophosphamide as GVHD prophylaxis and recent evidence suggesting the advantages of bone marrow as a preferred graft source in unrelated donor allo-HCT should also lead to decreased incidence and severity of chronic GVHD ^10, 11^.

GVHD remains one of the main complications after allo-HCT. Despite very intensive research, no major advances in the medical management of GVHD were made during the last two decades. Significant heterogeneity and a very complex pathogenesis of the disease as well as lack of well-defined specific therapeutic targets may explain this scarcity of proven success of novel drug therapies in patients with GVHD. However, over the last two decades, the decrease in frequency and severity of GVHD has led to a reduction in transplant-related mortality ^2^. This can be explained by changes in clinical practice associated with more effective prevention of severe acute and chronic GVHD. Selection of better-matched unrelated donors due to the use of high-resolution human leukocyte antigen (HLA) typing, increased use of non-myeloablative conditioning, and decreased use of total body irradiation in recent years can be associated with a reduction in the severity of acute GVHD ^9^. Increased frequency of post-transplant cyclophosphamide as GVHD prophylaxis and recent evidence suggesting the advantages of bone marrow as a preferred graft source in unrelated donor allo-HCT should lead to decreased incidence and severity of chronic GVHD ^10, 11^.

## A donor for all?

Strict HLA matching has been a requirement for allo-HCT for decades. Although HLA mismatching has been evaluated, the transplant outcomes have ranged from poor to suboptimal, depending on the degree of mismatch, because of substantially greater risk for GVHD and/or greater risk for infections owing to impaired immune reconstitution. Limited availability of suitable HLA-identical donors has been a significant barrier for allo-HCT eligibility for decades, particularly for minority populations ^12^. Wide acceptance of high-resolution donor-recipient HLA matching for HLA-A, -B, -C, and -DRB1 alleles was shown to be associated with improved survival after allo-HCT from unrelated donors by minimizing complications related to HLA mismatch ^13^. However, more accurate HLA typing also led to a new challenge: decreased availably of HLA-matched donors for a significant number of patients needing allo-HCT. For a long time, umbilical cord blood transplantation was the only feasible option for patients lacking a suitable HLA-matched donor; the immaturity of the newborn’s immunity allowed the use of mismatched cords without a greater risk of GVHD or rejection ^14^, but the small numbers of progenitors in the cord units make this option more difficult for many adults, and immune reconstitution is slower, putting the recipient at greater risk of transplant complication. New techniques for performing HCTs using haploidentical (half-matched) HCT (haplo-HCT) has changed the landscape. Historically, a high degree of HLA disparity between the donor and recipient in haplo-HCT was associated with unacceptably high rates of graft failure and GVHD and very poor survival. Several novel approaches are able to overcome the barriers of HLA disparity, which have led to significant improvement of haplo-HCT outcomes. The most widely used strategy for haplo-HCT is the administration of cyclophosphamide shortly after the infusion of stem cells in an attempt to eliminate alloreactive T cells (cells responsible for the development of GVHD) by sparing a majority of regulatory T cells (cells responsible for the development of graft versus tumor [GVT] activity) and hematopoietic stem cells ^15^.

Haplo-HCT followed by post-transplant cyclophosphamide has now become a widely used approach because of its simplicity, low transplant-related mortality, and low incidence of severe GVHD, even in older patients ^16^, and this has markedly expanded the pool of available donors ^17^. Some of the most recent data suggest that outcomes after haplo-HCT may be comparable to outcomes after fully HLA-matched unrelated donors ^18^, although there is concern that the risk of relapse may be higher.

## Reduction or delay of post-transplant relapse

Disease relapse remains the main challenge for both auto-HCT and allo-HCT. According to Center for International Blood and Marrow Transplant Research data, primary disease is responsible for almost two-thirds of deaths after auto-HCT ^19^. Relapse-related mortality is less common after allo-HCT because of GVT activity and a higher risk of early non-relapse mortality; however, it still remains the major cause of allo-HCT failure ^19^. The relapse risk can be decreased by inducing deeper responses to anti-tumor therapy prior to HCT ^20^, by enhancing the cytotoxic potential of a conditioning regimen ^21^, and by using maintenance therapy ^22^. In allo-HCT recipients, the relapse risk can be further decreased by enhancing the GVT effect.

Maintenance therapy with lenalidomide or bortezomib has led to a significant improvement in progression-free survival, depth of remissions ^23– 25^ and even overall survival ^24^ in patients with multiple myeloma (MM) who received auto-HCT. Now, maintenance therapy is an accepted standard of care for these patients. Several novel agents are currently being investigated for maintenance therapy in MM patients, and the results of these studies are expected shortly. An antibody-drug conjugate targeting CD30, brentuximab vedotin, was recently shown to significantly improve progression-free survival in patients with Hodgkin lymphoma with risk factors for relapse or progression after auto-HCT ^26^. In a recent multicenter randomized phase 3 trial, maintenance therapy with the anti-CD20 monoclonal antibody rituximab after auto-HCT was associated with a significantly increased survival rate among younger patients with mantle cell lymphoma ^27^.

## Future development

Many challenges remain, particularly in minimizing disease relapse and the severity of GVHD. Further research is needed to gain additional knowledge on how to enhance the ability of donor immune cells to eradicate malignant cells without significantly increasing GVHD. This will be possible with the development of novel adoptive immune cell and targeted therapies.

One of the drawbacks for clinicians has been the inability to predict the severity or treatment outcomes of GVHD. The severity of symptoms at the initial presentation of acute GVHD does not frequently predict its subsequent course, yet clinicians have historically determined both the intensity and the duration of immunosuppressive therapy largely based on the initial clinical presentation. Until recently, there were no laboratory tests accurately predicting the development and severity of acute GVHD. This situation has changed with the recent identification of GVHD biomarkers and the development and clinical testing of biomarker-based scoring algorithms to permit risk-adapted therapy for acute GVHD. Several serologic biomarkers such as TNFR1, ST2, and Reg3α have been identified and were recently validated to guide risk-adapted therapy at the onset of acute GVHD in a prospective multicenter study ^28^. These biomarkers can even predict the development of lethal GVHD when they are measured shortly after allo-HCT in patients without any clinical signs of GVHD ^29^. Therefore, the further development and validation of biomarker-based algorithms will enable clinicians to more quickly identify risks for acute GVHD so that customized treatment approaches can be used. This enables the use of less-intensive immunosuppressive approaches for better prognosis GVHD, sparing the risks of prolonged intensive immunosuppression; more-intensive treatment approaches for higher-risk GVHD require testing of new strategies to better improve control. Clinical trials are now underway to test this risk-adapted approach.

Recently, novel key targets and signaling pathways have been identified in the pathogenesis of chronic GVHD, including Bruton’s tyrosine kinase (BTK), Janus kinases (JAKs), spleen tyrosine kinase (SYK), and many others. These insights have led to clinical testing of the BTK inhibitor ibrutinib ^30^, several SYK inhibitors ^31^, and JAK1/2 inhibitors, including ruxolitinib ^32^, with very promising results in early studies. In a recent phase 2 study, the administration of ibrutinib showed a substantial response in up to two-thirds of patients with steroid-dependent or -refractory chronic GVHD ^30^, which has led to the breakthrough therapy designation of ibrutinib for chronic GVHD being granted by the US Food and Drug Administration.

Current evidence suggests the importance of commensal bacteria, particularly the gut microbiota, in influencing outcomes after allo-HCT, particularly its role in GVHD development and severity. Further research is urgently needed to better understand the relationship between gut microbiota and GVHD. Hopefully, novel therapies focusing on gut flora interventions may lead to more effective prevention and treatment of GVHD.

The implementation of immune-based therapies has a huge potential to reduce the risk of relapse after HCT. Despite encouraging efficacy of novel therapies such as chimeric antigen receptor (CAR) T-cell-based therapies and the use of bispecific antibodies and checkpoint inhibitors for a wide variety of malignancies, only very limited data are available for the utilization of such therapies after HCT. Because of their anti-tumor activity, all of these therapies hold significant promise in the prevention of treatment relapse after HCT, but their safety needs to be established before they are widely introduced into routine clinical practice. Currently, there is a growing body of evidence showing the efficacy and safety of these therapies after auto-HCT, but the risk of GVHD exacerbation, which can be fatal ^33^, after allo-HCT remains a concern.

Effective post-transplant therapies after allo-HCT are less established but urgently needed. Several post-transplant maintenance approaches after allo-HCT are currently being widely investigated. Maintenance therapy with a multi-kinase inhibitor, sorafenib, in patients with FMS-like tyrosine kinase-3-mutated acute myeloid leukemia (AML) ^22^ or low-dose azacitidine in patients with AML and myelodysplastic syndromes ^34^ have shown promising results but require confirmation in large clinical trials.

Immune escape is considered to be one of the key factors leading to relapse after allo-HCT, and there is considerable interest in developing novel therapies which are capable of enhancing the GVT effect. In one recent early-phase study, the administration of ipilimumab to induce CTLA-4 blockade led to clinically significant remissions in patients with disease relapse after allo-HCT without a significant increase in post-transplant complications ^35^. This observation suggests that other immune checkpoint inhibitors such as nivolumab or pembrolizumab can be used for the prevention and treatment of disease relapse after allo-HCT. Currently, several studies addressing this research question are being conducted.

Increasing use of cutting-edge molecular technologies such as genomic breakpoint cloning, single nucleotide polymorphism profiling, whole genome sequencing, whole exome sequencing, and RNA sequencing for prognostic, diagnostic, and therapeutic purposes is a very promising tactic for the management of HCT recipients. Further studies are required to better understand how to use these techniques to more accurately select patients for HCT as well as to more fully comprehend the biologic effects of HCT and select better targets of GVT responses.

Lastly, changes in transplantation practices with less-toxic conditioning regimens have allowed many patients of more advanced ages to be considered for HCT, and currently no strict upper age limit exists for this procedure. However, more studies are needed to better define the most optimal donor and patient selection and conditioning regimens, particularly for elderly patients with comorbidities.

## Abbreviations

Allo-HCT, allogeneic hematopoietic cell transplantation; AML, acute myeloid leukemia; auto-HCT, autologous hematopoietic cell transplantation; BTK, Bruton’s tyrosine kinase; CMV, cytomegalovirus; GVHD, graft versus host disease; GVT, graft versus tumor; haplo-HCT, haploidentical hematopoietic cell transplantation; HCT, hematopoietic cell transplantation; HLA, human leukocyte antigen; JAK, Janus kinase; MM, multiple myeloma; SYK, spleen tyrosine kinase.
